# Antifeedant Triterpenoids from the Seeds and Bark of *Lansium domesticum* cv Kokossan (Meliaceae)

**DOI:** 10.3390/molecules16042785

**Published:** 2011-03-29

**Authors:** Tri Mayanti, Roekmiati Tjokronegoro, Unang Supratman, Mat Ropi Mukhtar, Khalijah Awang, A. Hamid A. Hadi

**Affiliations:** 1 Department of Chemistry, Faculty of Mathematics and Natural Sciences, Padjadjaran University, Jatinangor 45363, Indonesia; 2 Department of Chemistry, Faculty of Science, University of Malaya, Kuala Lumpur 50603, Malaysia; E-Mails: matropi@um.edu.my (M.R.M.); khalijah@um.edu.my (K.A.); ahamid@um.edu.my (A.H.A.H.)

**Keywords:** kokosanolides, tetranortriterpenoid, onoceranoid, *Lansium domesticum*, meliaceae, antifeedant activity

## Abstract

Two tetranortriterpenoids, kokosanolide A (**1**) and C (**2**) were isolated from the seeds and three onoceranoid-type triterpenoids: kokosanolide B (**3**), 8,14-secogammacera-7,14-diene-3,21-dione (**4**) and a 1.5:0.5 mixture of 8,14-secogammacera-7,14(27)-diene-3,21-dione (**5)** and compound **4** were isolated from the bark of kokossan (*Lansium domesticum*). Complete ^1^H- and ^13^C-NMR data of the triterpenoids **1**-**5** are reported. The triterpenoids’ structures were elucidated primarily by means of high field 1D- and 2D-NMR, IR and HRMS spectral data. Triterpenoids **1**-**5** exhibited moderate to strong antifeedant activity against the fourth instar larvae of *Epilachna vigintioctopunctata*.

## 1. Introduction

*Lansium domesticum* cv Kokossan (family Meliaceae) is a higher tree, commonly called “kokosan’’ in Indonesia and widely distributed in Southeast Asian countries [1]. This plant has been reported to produce fruits and contain a bitter seed substance with antifeedant activity [2]. Previous phytochemical studies on *L. domesticum* reported the presence of tetranortriterpenoids [3,4,5], triterpenoid glycosides [6], onoceranoid-type triterpenoids [1] and onocerandiendione-type triterpenoids [5]. 

During the course of our continuing search for novel antifeedant compounds from tropical Meliaceae plants, the methanol extract of *L. domesticum* showed strong antifeedant activity against the fourth instar larvae of *Epilachna vigintioctopunctata*. Herein, we report the ^1^H and ^13^C NMR data and structural elucidation for these compounds **1**–**5** isolated from seed and bark extracts of the plant. The structures of compounds **1**, **3 **and **5** were established previously by X-ray diffraction [7,8,9]. 

**Figure 1:**
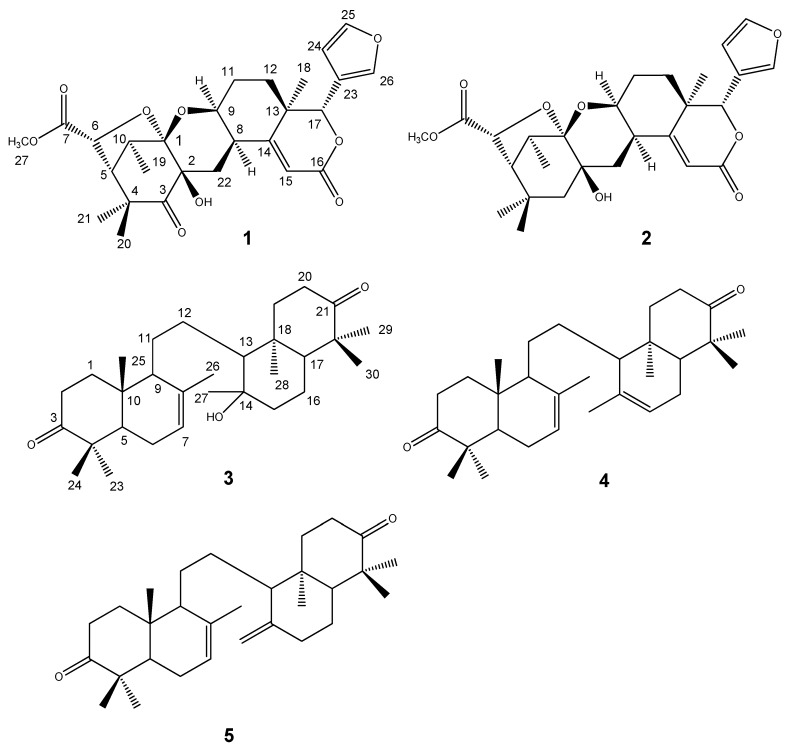
Structure of compounds **1–5**.

## 2. Results and Discussion

Liquid-liquid partitioning of the MeOH extract of the seed of *L. domesticum* cv Kokossan into *n*-hexane, EtOAc and aqueous MeOH fractions gave the *n*-hexane fraction (4 g) as the most active one, with 100% antifeedant activity at 1% concentration. Purification of the *n*-hexane fraction using silica gel 60 open column chromatography led to the isolation of compounds **1** and **2**. In addition, the MeOH extract of the bark of *L. domesticum* was partitioned between *n*-hexane and ethyl acetate to give the ethyl acetate fraction. A crude ethyl acetate fraction was subjected to vacuum column chromatography on silica gel 60 and further purified by silica gel column chromatography to yield compounds **3**–**5**. 

**Figure 2:**
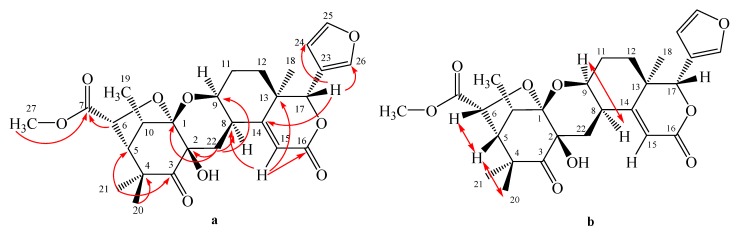
Selected HMBC (H→C) (**a**) and NOESY (↔) (**b**) correlations of kokosanolide A.

Kokosanolide A (**1**) was obtained as a white needle-like crystals, m.p. 178–180 °C, from *n*-hexane-EtOAc. Its molecular formula was established to be C_27_H_32_O_9_ by LC-ESI-MS data (*m/z* 500.8093, [M+H]^+^), which combined with the ^1^H- and ^13^C-NMR spectral data (Table 1), thus indicated 12 degrees of unsaturation. The UV spectrum showed an absorption maximum at 282 nm (ε 4,600), indicating the presence of an α-β-unsaturated ketone. The IR spectrum showed bands which were ascribable to hydroxyl (ν_max_ 3,427 cm^−1^), a ketone (ν_max_ 1,753 cm^−1^), unsaturated ketone (ν_max_ 1,709 cm^−^^1^), isolated double bond (ν_max_ 1,631 cm^−1^) and *gem*-dimethyl (ν_max_ 1,449 and 1,389 cm^−1^) functionalities. The ^1^H-NMR spectrum showed the presence of three singlets (δ 0.98, 1.07 and 1.37) from tertiary methyl groups and one doublet at δ 1.13 (*J *= 8 Hz) from a secondary methyl group which in turn was correlated to H-10 (δ 3.33, 1H, q, *J *= 8 Hz). A singlet appeared in the downfield region (δ 3.67) that was ascribed to the C-27 methoxy protons. A detailed analysis of ^1^H-NMR spectrum showed characteristic signals of a tetranortriterpenoid skeleton with a *β*-substituted furan at δ 7.46, 7.40 and 6.43 and the presence of an olefinic signal of an *α,β*-unsaturated ketone at δ 6.28 (1H, s) [3,4]. The ^13^C-NMR, together with APT spectra, revealed 27 carbon signals, including characteristic signals due to a furan ring [δ 143.5 (d), 141.9 (d), 120.5 (s) and 110.8 (d)], a ketone (δ 208.5), two ester groups (δ 172.4 and 165.4), one oxygenated carbon (δ 108.4) and *α*,*β*-unsaturated ketone (δ 168.6 and 116.7), thus suggesting that **1** possesed a hexacyclic structure with a furan moiety. The ^1^H-^1^H COSY spectrum of **1** showed proton correlations of H_5_/H_6_, H_9_/H_8_/H_22_, H_9_/H_11_/H_12_, H_5_/H_10_/H_19_, and H_24_/H_25_, supporting the presence of tetranortriterpenoid structure with a furan ring [3,4,5]. The connectivity of these partial structures were established from the HMBC spectral data (Figure 2). 

The oxygenated H-17 (δ 5.17) revealed correlations to C-23 (δ 120.5), C-24 (δ 110.8) and C-26 (δ 141.9), indicating that the furan ring was located at C-17. The signals correlating H-15 (δ 6.28) with C-8 (δ 34.6), C-13(δ 39.6), and C-16 (δ 165.4), suggested the presence of an α,β-unsaturated δ-lactone ring system. The positioning of the pyran ring was established by the correlation signals of H-22 (δ_H_ 2.46 and 2.77) with C-1 (δ 108.4), C-2 (δ 76.1), C-9 (δ 69.5), C-8 (δ 34.6) and C-14 (δ 168.6).

**Table 1:** NMR spectral data for compounds **1 **and **2**.^ a^

1				2	
Position	^13^C NMR	^1^H NMR	HMBC	^13^C NMR	^1^H NMR
	δ_C_ (mult., ppm)	δ_H_ (integral, mult., *J* Hz)	(^1^H to ^13^C)	δ_C_ (mult., ppm)	δ_H_ (integral, mult., *J* Hz)
1	108.4 (s)	-	-	106.9 (s)	-
2	76.1 (s)	-	-	76.1 (s)	-
3	208.6 (s)	-	-	20.9 (t)	1.71 (1H, d, 6.2)
					2.18 (1H, m)
4	48.4 (s)	-	-	47.8 (s)	-
5	56.4 (d)	2.27 (1H, dd, 4, 7)	1, 3, 4, 10	55.9 (d)	2.12 (1H, dd, 3.7, 6)
6	77.4 (d)	4.81 (1H, d, 4)	4, 5, 7	76.7 (d)	4.82 (1H, d, 4)
7	172.4 (s)	-	-	171.9 (s)	-
8	34.6 (d)	2,64 (1H, dd, 5.3, 6.5)	14	34.4 (d)	2.31 (1H, m)
9	69.5 (d)	4.39 (1H, m)	14	67.8 (d)	4.13 (1H, m)
10	37.3 (d)	3.33 (1H, q, 8)	2, 4, 5, 19	36.5 (d)	3.29 (1H, m)
11	25.3 (t)	1.79 (1H, m)	8, 12, 13	26.8 (t)	1.76 (1H, m)
		1.91 (1H, m)	-		1.90 (1H, m)
12	27.8 (t)	1.26 (1H, m)	9, 11, 13, 17	29.4 (t)	1.23 (1H, m)
		1.74 (1H, m)	-		1.72 (1H, m)
13	39.6 (s)	-	-	38.4 (s)	-
14	168.6 (s)	-	-	167.4 (s)	-
15	116.7 (d)	6.28 (1H, s)	8, 13, 16	117.4 (d)	6.43 (1H, s)
16	165.4 (s)	-	-	165.4 (s)	-
17	81.5 (d)	5.17 (1H, s)	14, 23, 24, 26	81.7 (d)	5.14 (1H, s)
18	18.2 (q)	1.07 (3H, s)	12, 13, 14, 17	19.6 (q)	1.26 (3H, s)
19	12.2 (q)	1.13 (3H, d, 8)	1, 5, 10	11.7 (q)	1.17 (3H, d, 7.8)
20	23.7 (q)	0.98 (3H, s)	3, 4, 5, 21	23.3 (q)	0.98 (3H, s)
21	30.1 (q)	1.37 (3H, s)	3, 4, 5, 20	30.9 (q)	1.37 (3H, s)
22	25.4 (t)	2.46 (1H, dd, 7.5, 5.3)	1, 2, 3, 8, 9, 14	25.7 (t)	2.41 (1H, m)
		2.77 (1H, dd, 7.5, 4.7)	-	-	2.70 (1H, m)
23	120.5 (s)	-	-	119.7 (s)	-
24	110.8 (d)	6.43 (1H, d, 4.4)	23, 25, 26	110.2 (d)	6.45 (1H, s)
25	143.5 (d)	7.40 (1H, d, 4.4)	23, 24, 26	142.8 (d)	7.40 (1H, s)
26	141.9 (d)	7.47 (1H, s)	23, 24, 25	141.4 (d)	7.47 (1H, s)
27-OCH_3_	52.6 (q)	3.67 (3H, s)	-	52.2 (q)	3.68 (3H, s)

^a^ Taken in CDCl_3_ at 500 MHz for ^1^H and at 125 MHz for ^13^C.

**Figure 3:**
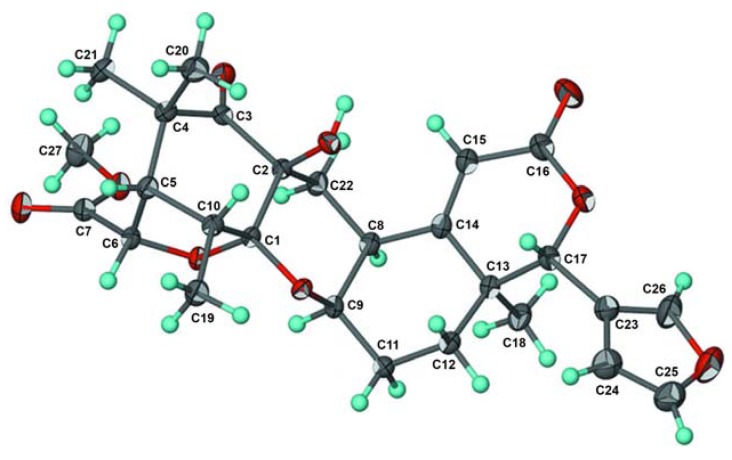
ORTEP drawing for kokosanolide A reproduced from Mayanti *et al.* [7].

Other correlations of methyl signal at δ 0.98 and 1.37 to C-3 (δ 208.6), C-4 (48.4) and C-5 (δ_H_ 56.4), suggesting that *gem*-dimethyl was located at C-4. The carbomethoxyl signal (δ 3.67) and the signal of H-6 (δ 4.81) were correlated to an ester carbonyl (δ 172.4), suggesting that an ester group was located at C-6. The relative configuration of **1** was elucidated by NOESY correlations as shown in Figure 2. NOESY correlation of H-8/H-9 suggesting that pyran ring should be β−orientation. Correlations between H-6/H-5/H-20 indicated that methyl group and tetrahydrofuran ring should be α-orientation. Thus, the gross structure of tetranortriterpenoid **1** was elucidated as a hexacyclic ring system. The structure and relative stereochemistry were further elucidated by using single-crystal X-ray diffraction analysis [7]. An ORTEP drawing of **1** is shown in Figure 3. Consequently, the structure of tetranortriterpenoid **1** was established to be a tetranortriterpenoid and was named kokosanolide A. 

Kokosanolide C (**2**) was obtained as colorless needle-like crystals from *n*-hexane-EtOAc and decomposed during the measurement of its melting point. The UV spectrum showed an absorption maximum at 275 nm (ε 4,500), indicating the presence of an α-β-unsaturated ketone. The IR spectrum showed bands which were ascribable to hydroxyl (ν_max_ 3,563 cm^−1^), a ester carbonyl (ν_max_ 1,758 cm^−1^) and unsaturated ketone (ν_max_ 1,704 cm^−^^1^). The ^1^H- and ^13^C-NMR (Table 1) spectra of **2** were quite similar to those of **1**, except for the absence of the ketone signal at δ 208.5 and appearance of a geminal proton signal at [δ_H_ 2.18 (1H, m), 1.71 (1H, m); δ_C_ 20.9]. In the HMBC spectrum of **2**, long range correlations were observed between the signals at δ 1.72 and 2.18 and the carbon signals at δ 76.1 (C-2), 47.8 (C-4) and 55.9 (C-5), suggesting that compound **2** was a 3-deoxo derivative of compound **1** and it was thus named kokosanolide C.

**Figure 4:**
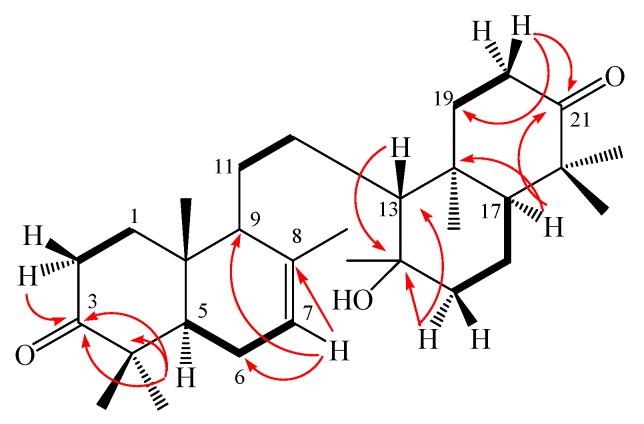
Selected HMBC (H→ C) and COSY ( ▬) correlations for kokosanolide B.

**Figure 5:**
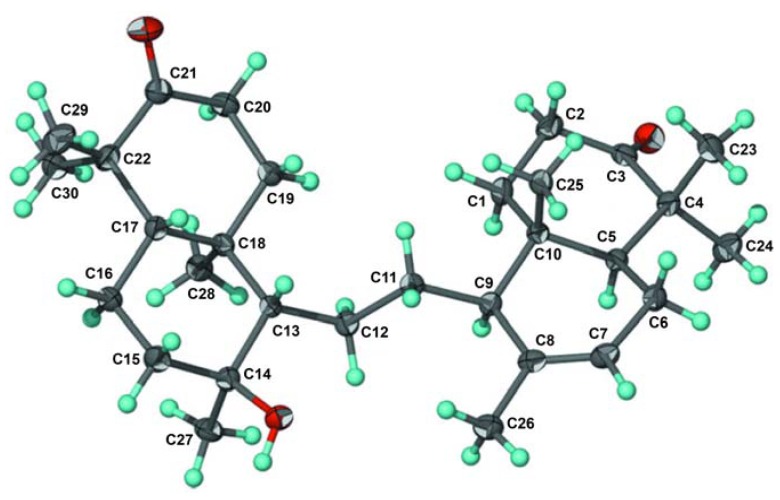
ORTEP drawing for kokosanolide B reproduced from Supratman *et al.* [8].

Kokosanolide B (**3**) was obtained as cubic crystals, m.p. 148–150 °C, from *n*-hexane-EtOAc. The molecular formula of **3** was determined to be C_30_H_48_O_3_ by LC- ESI-MS data (*m/z* 456.6892, [M+H]^+^), and combined with the ^1^H- and ^13^C-NMR spectral data (Table 2), thus required seven degrees of unsaturation. IR absorption bands at 3,749, 1,705, 1,384 and 1,261 cm^−1^ suggested the presence of hydroxyl, carbonyl, and *gem*-dimethyl functionalities, respectively. Analysis of ^1^H- and ^13^C-NMR data, DEPT and the HMQC spectra of **3** revealed the presence of thirty signals: three sp^2^ and five sp^3^ quaternary carbons, one sp^2^ and four sp^3^ methines, nine sp^3^ methylenes, and eight methyl groups. Among them, one sp^2^ methine (δ_C_ 121.7; δ_H_ (1H, 5.41, m) was ascribed to the isolated double bond, while two carbonyl carbons (δ 216.9 and 217.1 ppm) and one sp^3^ carbon (δ 74.0) were assigned to those bearing an oxygen atom (C-3, C-21 and C-14, respectively), suggesting that compound **3 **was an onoceranoid-type triterpenoid [5]. The positions of the ketones, hydroxyl and isolated double bond were further determined by the COSY and HMBC experiments (Figure 4), H-7 (δ 5.41) showed correlations to C-6 (δ 28.9), C-9 (δ 55.5) and C-8 (δ 135.3), suggesting that an isolated double bond is located at C-7 and C-8. The methylene signals at C-2 (δ 2.23) revealed correlations to carbonyl signal at δ 216.9, whereas H-5 (δ 1.57) was correlated to C-4 (δ 47.6) and C-3 (δ 216.9), indicating that one of the carbonyl moeity and *gem*-dimethyl are placed at C-3 and C-4, respectively. Furthermore, the methylene signals at C-20 (δ 2.26) showed correlations to the carbonyl signal at δ 217.1, whereas the methine signal at C-17 (δ 1.42) showed correlations to C-22 (δ 47.6 ) and C-21 (δ 217.1), thus suggesting the other carbonyl group and *gem*-dimethyls were located at C-21 and C-22, respectively. An oxygenated tertiary carbon signal was revealed to be C-14 by the correlation between methine signal at C-13 (δ_C_ 1.12 ) to oxygenated carbon at δ_C_ 74.0 ppm and correlation between methylene signals at C-15 (δ_C_ 1.46 ) to oxygenated carbon at δ_C_ 74.0 ppm. Thus, the structure of onoceranoid-type triterpenoid **3** was determined as 8,14-secogammacera-14-hydroxy-7-ene-3,21-dione and was named kokosanolide B. The structure and relative stereochemistry were also elucidated by using single-crystal X-ray diffraction analysis [8]. An ORTEP drawing of **3** is shown in Figure 5.

**Table 2:** NMR spectral data for compound **3 **and **4**.^ a^

	3			4	
Position	^13^C NMR	^1^H NMR	HMBC	^13^C NMR	^1^H NMR
	δ_C_ (mult., ppm)	δ_H_ (integral, mult., *J* Hz)	(^1^H to ^13^C)	δ_C_ (mult., ppm)	δ_H_ (integral, mult., *J* Hz)
1	38.5 (t)	1.91 (1H, m); 2.08 (1H, m)	2, 3, 10	38.5 (t)	1.46 (1H, m); 2.09 (1H, m)
2	34.8 (t)	2.23 (1H, m); 2.41 (1H, m)	3	34.8 (t)	2.24 (1H, m); 2.70 (1H, m)
3	216.9 (s)	-	-	216.9 (s)	-
4	47.6 (s)	-	-	47.6 (s)	-
5	51.6 (d)	1.57 (1H, m)	9, 10, 24	51.6 (d)	1.59 (1H, dd, 5, 7)
6	28.9 (t)	1.12 (1H, m); 2.56 (1H, m)	7, 10	30.1 (t)	1.33 (1H, dd, 7, 10); 1.24 (1H, dd, 5, 10)
7	121.7 (d)	5.41 (1H, m)	8	122.1 (d)	5.43 (1H, m)
8	135.3 (s)	-	-	135.3 (s)	-
9	55.5 (d)	1.59 (1H, m)	26	55.6 (d)	1.65 (1H, m)
10	36.6 (s)	-	-	36.7 (s)	-
11	21.5 (t)	1.61 (1H, m); 2.41 (1H, m)	9	24.2 (t)	1.93 (1H, m); 2.40 (1H, m)
12	21.5 (t)	1.62 (1H, m); 1.76 (1H, m)	9	24.2 (t)	1.93 (1H, m); 2.40 (1H, m)
13	61.8 (d)	1.12 (1H, m)	17	55.6 (d)	1.65 (1H, m)
14	74.0 (s)	-	-	135.3 (s)	-
15	44.2 (t)	1.46 (1H, m); 2.23 (1H, m)	14, 17	122.1 (d)	5.43 (1H, m)
16	31.4 (t)	1.51 (1H, m); 1.84 (1H, m)	17	30.1 (t)	1.33 (1H, dd, 7, 10); 1.24 (1H, dd, 5, 10)
17	55.2 (d)	1.42 (1H, m)	18, 22	51.6 (d)	1.59 (1H, dd, 5, 7)
18	36.6 (s)	-	-	36.7 (s)	-
19	38.4 (t)	1.78 (1H, m); 2.10 (1H, m)	21	38.5 (t)	1.46 (1H, m); 2.09 (1H, m)
20	34.1 (t)	2.26 (1H, m); 2.73 (1H, m)	19, 21	34.8 (t)	2.24 (1H, m); 2.70 (1H, m)
21	217.1 (s)	-	-	216.9 (s)	-
22	47.6 (s)	-	-	47.6 (s)	-
23	25.1 (q)	1.04 (3H, s)	5, 24	25.1 (q)	1.04 (3H, s)
24	22.3 (q)	1.08 (3H, s)	23	22.3 (q)	1.09 (3H, s)
25	13.4 (q)	0.96 (3H, s)	5, 9, 10	13.5 (q)	0.97 (3H, s)
26	22.3 (q)	1.77 (3H, s)	9	22.5 (q)	1.72 (3H, s)
27	24.2 (q)	1.21 (3H, s)	13, 14, 15	22.5 (q)	1.72 (3H, s)
28	15.1 (q)	0.93 (3H, s)	13, 17	13.5 (q)	0.97 (3H, s)
29	21.4 (q)	1.02 (3H, s)	17, 22, 30	25.1 (q)	1.04 (3H, s)
30	26.5 (q)	1.09 (3H, s)	17, 29	22.3 (q)	1.09 (3H, s)

^a^ Taken in CDCl_3_ at 500 MHz for ^1^H and at 125 MHz for ^13^C.

8,14-Secogammacera-7,14-diene-3,2-dione (**4**) was obtained as a white needle-like crystals, m.p. 143–144 °C, from *n*-hexane-EtOAc. The molecular formula of **4** was determined to be C_30_H_46_O_2_ by LC-ESI-MS data (*m/z* 438.3745, [M+H]^+^), which together with ^1^H- and ^13^C-NMR spectral data (Table 2), requires eight degrees of unsaturation. Compound **4** showed no absorption maxima in the UV spectrum indicating the absence of a conjugated double bond. The IR spectrum showed bands which were ascribed to a ketone (ν_max_ 1,708 cm^−1^), isolated double bond (ν_max_ 1,662 cm^−1^) and *gem*-dimethyl (ν_max_ 1,430 and 1,360 cm^−1^). The ^13^C-NMR spectrum of **4** showed 15 signals, similar to those of kokosanolide B, suggesting that **4** has a symmetrical structure. The essential differences between the NMR spectra of **4** and kokosanolide B consisted of the absence of a hydroxyl group and appearance of a double bond [δ 5.43 (1H, m), δ_H_ 122.1 and 135.3] and fifteen carbon signals, suggesting that **4** was a dehydroxy derivative of kokosanolide B. In order to determine the connectivity of the partial structure due to a newly double bond, HMBC experiments were carried out. The signal of olefinic proton H-15 (δ 5.43) was correlated to C-14 (δ 135.3), C-13 (δ 55.6) and C-16 (δ 30.1), indicating that a new double bond was located at C-14 and C-15, suggesting that **4 **has two similar unit structure. Consequently, compound **4** was identified as a 8,14-secogammacera-7,14-diene-3,21-dione [5].

**Figure 6:**
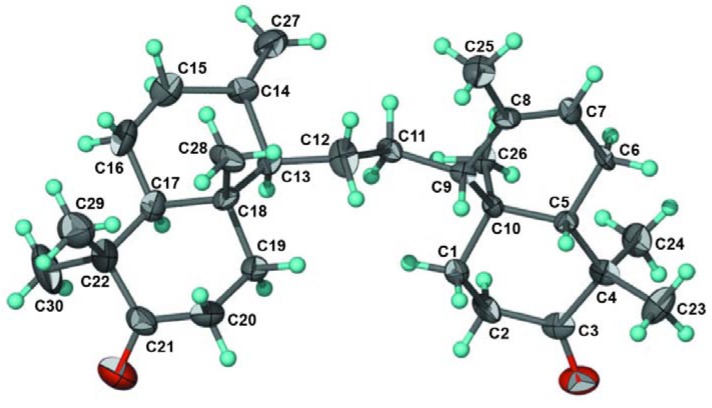
ORTEP drawing for **5** reproduced from Tjokronegero *et al. *[9].

Compound (**5**) was identified as 8,14-secogammacera-7,14(27)-diene-3,21-dione. It was isolated together with compound **4** with a ratio of 1.5:0.5. The ^1^H and ^13^C-NMR spectra of **5** were similar to those of **4**, except for the appearance of methylene protons and an sp^2^ carbon at C-27 [δ_H_ 5.12 (1H, *J *= 10.5 Hz), 5.45 (1H, *J *= 10.5 Hz), δ_C_ 122.1], indicating that **5** is an isomer of **4**. The structure was elucidated by using a single-crystal X-ray diffraction analysis [9] and the ORTEP drawing for **5** is shown in Figure 6 below. 

Compounds **1**–**5** were evaluated for antifeedant activity against the fourth instar larvae of *Epilachna vigintioctopunctata* at a concentration of 1%. The antifeedant activities of compounds **1**–**5** are shown in Table 3. Among those compounds, kokosanolide C (**2**), lacking the ketone group, showed less antifeedant potency, whereas 8,14-secogammacera-14-hydroxy-7-ene-3,21-dione (**3**) having the hydroxyl group showed the strongest activity, thus, suggested that an oxygenated fuctional group was an important structural component for antifeedant activity.

**Table 3:** Antifeedant activity of compounds **1**–**5**.

Compound	Activity (%)
Kokosanolide A (**1**)	78
Kokosanolide C (**2**)	0
Kokosanolide B (**3**)	99
8,14-Secogammacera-7,14-diene-3,21-dione (**4**)	85
8,14-Secogammacera-7,14(27)-diene-3,21-dione(**5**)	56

## 3. Experimental

### 3.1. General

Melting points were measured on an Electrothermal melting point apparatus and are uncorrected. Optical rotations were recorded on a Perkin-Elmer 341 polarimeter. The UV spectra were obtained on a UV Ultraspec 3000 Pro spectrophotometer. The IR spectra were recorded on a Perkin-Elmer 1760X FT-IR in KBr. The mass spectra were recorded with a Mariner Biospectrometry-Finnigan instrument. ^1^H- and ^13^C-NMR spectra were obtained with a JEOL JNM A-500 spectrometer using TMS as internal standard. All ORTEP diagrams were obtained from previous reports. Chromatographic separations were carried out on silica gel 60 (Merck). TLC plates were precoated with silica GF_254_ (Merck, 0.25 mm) and detection was achieved by spraying with 10% H_2_SO_4_ in ethanol, followed by heating. 

### 3.2. Plant material

The bark and seed of *L. domesticum* cv Kokossan were collected in Cililin District, Bandung, West Java Province, Indonesia in March 2006. The plant was identified by the staff of the Laboratory of Plant Taxonomy, Department of Biology, Padjadjaran University, Indonesia. A voucher specimen (No. 10184) was deposited at the herbarium of the Padjadjaran University.

### 3.3. Antifeedant bioassay

Compounds **1**–**5** at a concentration of 1% were subjected to select antifeedant assays on *Solanum nigrum* leaves against 4th instars larvae of *Epilachna vigintioctopunctata*. Antifeedant activity (%AF) was determined by using the equation %AF = (1 – treatment consumption/control consumption) × 100 [11]. The antifeedant activities of **1**–**5** at 1% concentration are presented in Table 3. 

### 3.4. Extraction and isolation

Dried seeds of *L. domesticum *cv kokossan (2 kg) were extracted exhaustively with methanol 12 L at room temperature for 3 days. The resulting methanol extract (84 g) was partitioned between *n*-hexane (2.5 L) and 10% aqueous methanol (2.5 L) to give an *n*-hexane soluble fraction (4 g) after removal of the solvent. The *n*-hexane extract was subjected to column chromatography on silica gel 60 using a *n*-hexane and dichloromethane (8:2). The fraction eluted with *n-*hexane-dichloromethane (6:4) was further separated by column chromatography on silica gel (*n*-hexane-ethyl acetate 7:3) to give **1** (150 mg) and **2** (26 mg). 

The dried bark of *L. domesticum *cv kokossan (3 kg) was extracted exhaustively with methanol 15 L at room temperature for 3 days. The methanol extract (250 g) was partitioned with *n*-hexane (3 L) and ethyl acetate (3 L) to give an *n*-hexane soluble fraction (70 g) and an ethyl acetate soluble fraction (40 g). The ethyl acetate fraction was subjected to vacuum column chromatography on silica gel 60 by using a step gradient of *n*-hexane/ethyl acetate. The fraction eluted with *n*-hexane:ethyl acetate (80:20) was further separated by column chromatography on silica gel using *n*-hexane:ethyl acetate (95:5) to yield an active fraction (1.5 g). The active fraction was further chromatographed on silica gel using *n*-hexane/acetone (90:10) to give **3** (10 mg), **4** (50 mg) and **5** (24 mg).

*Kokosanolide A*** (1**). White needle-like crystals; [α]^20^_D_ + 85° (*c* 0.5, CHCl_3_); UV (λ_max_ in EtOH) 282 nm (ε 4,600); IR (KBr) ν_max_ 3,427, 1,753, 1,709, 1,631, 1,449 and 1,389 cm^−1^. ^1^H-NMR (CDCl_3_, 500 MHz), see Table 1; ^13^C-NMR (CDCl_3_, 125 MHz), see Table 1; LC-ESI-MS data (*m/z* 500.8093, [M+H]^+^).

*Kokosanolide C* (**2**). Colorless needle-like crystals; [α]^20^_D_ + 96° (*c* 0.5, CHCl_3_); UV (λ_max_ in EtOH) 275 nm (ε 4,500); IR (KBr) ν_max_ 3,563 cm^−1^, 1,758 cm^−1^, 17,04 cm^−1^. ^1^H-NMR (CDCl_3_, 500 MHz), see Table 1; ^13^C-NMR (CDCl_3_, 125 MHz), see Table 1.

*Kokosanolide B* (**3**). cubic crystals; [α]^20^_D_ – 18.5° (*c* 01.0, MeOH); IR (KBr) ν_max_ 3,749, 1,705, 1,384 and 1,261 cm^−1^. ^1^H-NMR (CDCl_3_, 500 MHz), see Table 2; ^13^C-NMR (CDCl_3_, 125 MHz), see Table 2. LC-ESI-MS data (*m/z *456.6892, [M+H]^+^).

*8,14-Secogammacera-7,14-diene-3,21-dione* (**4**). Needle-like crystals; [α]^20^_D_ − 8° (*c* 0.5, MeOH); IR (KBr) ν_max_ 1,708, 1,662, 1,430, 1,360 cm^−1^. ^1^H-NMR (CDCl_3_, 500 MHz), see Table 2; ^13^C-NMR (CDCl_3_, 125 MHz), see Table 2. LC-ESI-MS data (*m/z* 438.3745, [M+H]^+^).

*8,14-Secogammacera-7,14(27)-diene-3,21-dione* (**5**). Needle-like crystals; IR (KBr) ν_max_ 1,667, 1,454 and 1,384 cm^−1^. ^1^H-NMR (CDCl_3_, 500 MHz), δ_H_ (ppm), 0.95 (3H, s), 0.97 (3H, s), 1.04 (3H, s), 1.08 (3H, s), 1.09 (3H, s), 1.10 (3H, s), 1.20 (1H, m), 1.22 (3H, s), 1.24 (1H, dd, *J *= 5, 10 Hz), 1.33 (1H, dd, *J *= 7, 10 Hz), 1.40 (1H, m), 1.46 (1H, m), 1.50 (1H, m), 1.59 (1H, dd, *J *= 5, 7 Hz), 1.63 (1H, m), 1.65 (1H, m), 1.72 (3H, s), 1.79 (1H, m), 1.83 (1H, m), 1.93 (1H, m), 2.04 (1H, m), 2.09 (1H, m), 2.15 (1H, m), 2.24 (1H, m), 2.35 (1H, m), 2.40 (1H, m), 2.45 (1H, m), 2.70 (1H, m), 5.12 (1H, *J *= 10.5 Hz), 5.43 (1H, m), 5.45 (1H, *J *= 10.5 Hz); ^13^C-NMR (CDCl_3_, 125 MHz), δ_C_ (ppm); 13.5, 22.3, 22.5, 24.2, 25.1, 30.1, 34.8, 34.9, 36.7, 38.5, 47.6. 51.2, 51.6, 55.6, 122.1, 135.3, 216.9, 217.0.

## 4. Conclusions

The methanolic extract from the dried seed and bark of *Lansium domesticum* cv kokossan yield two tetranortriterpenoids – kokosanolide A and C – and one onoceranoid-type triterpenoid, kokosanolide B, along with two onoceranoid-type triterpenoids; 8,14-secogammacera-7,14-diene-3,21-dione and a mixture of 8,14-secogammacera-7,14(27)-diene-3,21-dione and 8,14-secogammacera-7,14-diene-3,21-dione (1.5:0.5). The results of this study provide a basis for futher phytochemical studies on the *Lansium* plants. This study also suggests that the *Lansiu*m plants contain potent antifeedant compounds for further application.
